# How Long Do People With Arthritis Stay Healthy and in Work? Analysis of Data From the Health and Retirement Study

**DOI:** 10.1002/acr2.70142

**Published:** 2026-01-20

**Authors:** Ross Wilkie, Jessica Potts, Oluwasikemi Onamusi, Glenn Pransky, Marty Lynch

**Affiliations:** ^1^ School of Medicine, Keele University Staffordshire United Kingdom; ^2^ Harvard T.H. Chan School of Public Health Boston Massachusetts

## Abstract

**Objective:**

To estimate healthy working life expectancy (HWLE; the average number of years that adults from age 50 years can expect to be healthy and in paid work) in the United States for people with arthritis overall and by sex, education, ethnicity, region, and obesity status.

**Methods:**

Longitudinal survey data (14 waves of the Health and Retirement Study) for adults aged ≥50 years were used. “Healthy” and “working” were defined as no limiting long‐standing illness and employment/self‐employment, respectively. Age‐adjusted, continuous‐time, five‐state models were fitted using Interpolated Markov Chains ( IMaCh) software, a maximum likelihood modeling program using interpolation of Markov Chains, to estimate hazards of transitions out of the healthy and working state based on arthritis status (and the covariates sex, ethnicity, region, education, and obesity status).

**Results:**

Weighted values are for 37,062 participants across 14 waves. HWLE at age 50 years for people with arthritis was almost half that for those without arthritis (6.18 years [95% confidence interval (CI) 6.06–6.30 years] vs 11.71 years [95% CI 11.56–11.86 years]). Those with both obesity and arthritis had the smallest HWLE values at age 50 years.

**Conclusion:**

The extent of reduction in a healthy and working state highlights the societal impact of arthritis. Variation in the amount of time in a healthy and working state by sociodemographic factors suggests that there are approaches that can increase HWLE. Further exploration of workplace factors and employment opportunities may lead to strategies to reduce the impact of arthritis on healthy working lives.

## INTRODUCTION

There is increasing attention on extending working lives for people with arthritis.^1^ The high and increasing prevalence of arthritis in adults from age 50 years onward indicates that arthritis will have an increasing impact on work.^2^, ^3^ In the United States, in line with population aging, the age of the working population is increasing, with employment levels in workers older than 65 years doubling.^1^ People are choosing to work until they are older, sometimes for financial needs but also because work can be good for maintaining physical, mental, and social health and aligning with an individual's sense of purpose.^4^ With people working until they are older, resulting in increasing prevalence of workers with arthritis, there will be increasing demand on health care and employers to support workers to remain in work.^5^, ^6^ Capturing the ability of populations and those with arthritis to extend working lives is important for identifying potential demand on health care and employers to manage health‐related work issues and the development of employment opportunities.^6^

**SIGNIFICANCE & INNOVATIONS**

- This article uses nationally representative data to estimate the amount of time that people in the United States with arthritis are healthy and in work.
- The amount of time that people in the United States with arthritis at age 50 years are healthy and in work is on average almost half that for those without arthritis (6.18 years [95% confidence interval (CI) 6.06–6.30 years] vs 11.71 years [95% CI 11.56–11.86 years]).
- This article identifies that the amount of time that people with arthritis are healthy and in work differs by sex, educational attainment, ethnicity, and where people live.
- This article highlights the significantly larger amount of time of healthy working life lost in people with arthritis compared to those with obesity.

Healthy working life expectancy (HWLE) is a measure of the average number of years that populations are healthy and in work^7^, ^8^; the higher the HWLE, the more able populations are to extend their working lives. HWLE and the potential to extend working lives is dependent on health, demographics, lifestyle, and workplace factors.^9^ In this study, we examine the role of obesity as an example of a common comorbidity with arthritis, which is associated with incident arthritis and work disability predominantly through physical health issues.^10^, ^11^, ^12^, ^13^

There are no current estimates of HWLE for people with arthritis in the United States. This study used data from the Health and Retirement Survey (HRS), a nationally representative sample of adults aged 50 years and older in the United States, to estimate HWLE. We aim to provide estimates for the overall population of the United States and by sex, educational attainment, ethnicity, and region. We also estimate the levels of HWLE for people with arthritis and obesity and summarize the average amount of time lost linked to these conditions.

## METHODS

### Data and study sample

Full details of the study design, methods, and ethical approval of the HRS have been published (https://hrs.isr.umich.edu/about); this ethical approval means that this study did not require additional approval. The HRS is a longitudinal survey of a large sample of US adults aged >50 years, with results weighted to reflect the overall US population. Data were used from each HRS biennial survey time point between 1994 and 2020 (14 time points). Information was obtained for interview date, survey participation, health status, work status, and status for the time‐varying covariates arthritis and obesity (classified using body mass index [BMI] calculated from bodyweight at each wave). HRS baseline (or other first) was used for identification of sex, ethnicity, address region, and education as fixed (time‐invariant) covariates, as well as date of birth and height (to calculate BMI and classify obesity). Death dates from HRS exit interview data (1996–2020) were used to censor the vital status of participants without recorded death dates for time points subsequent to their latest interviews.

We identified a sample of cohort‐eligible, community‐dwelling study participants aged 50 years and older; we assigned each participant a longitudinal survey weight taken as the first non‐zero cross‐sectional HRS survey weight for a survey taken while aged 50 years or older throughout the study period (1994–2020) using data from the HRS cross‐wave tracker file (release 2020 V1.0).

### Assessment of health and work

Health and working status were self‐reported in each survey wave. “Healthy” was defined as no limitation in activities due to a health problem. Participants were identified as healthy if they did not report limitation in any of the following three questions: (1) “Do you have any impairment or health problem that limits the kind or amount of paid work you can do?” (2) “Does any impairment or health problem limit the kind or amount of work you can do around the house?” and (3) “Are you limited in any way in activities because of an impairment or problem?” Those who indicated yes to one or more questions were identified as unhealthy. Data were classed as missing if a participant did not respond to any of the activity limitation questions.

**Figure 1:**
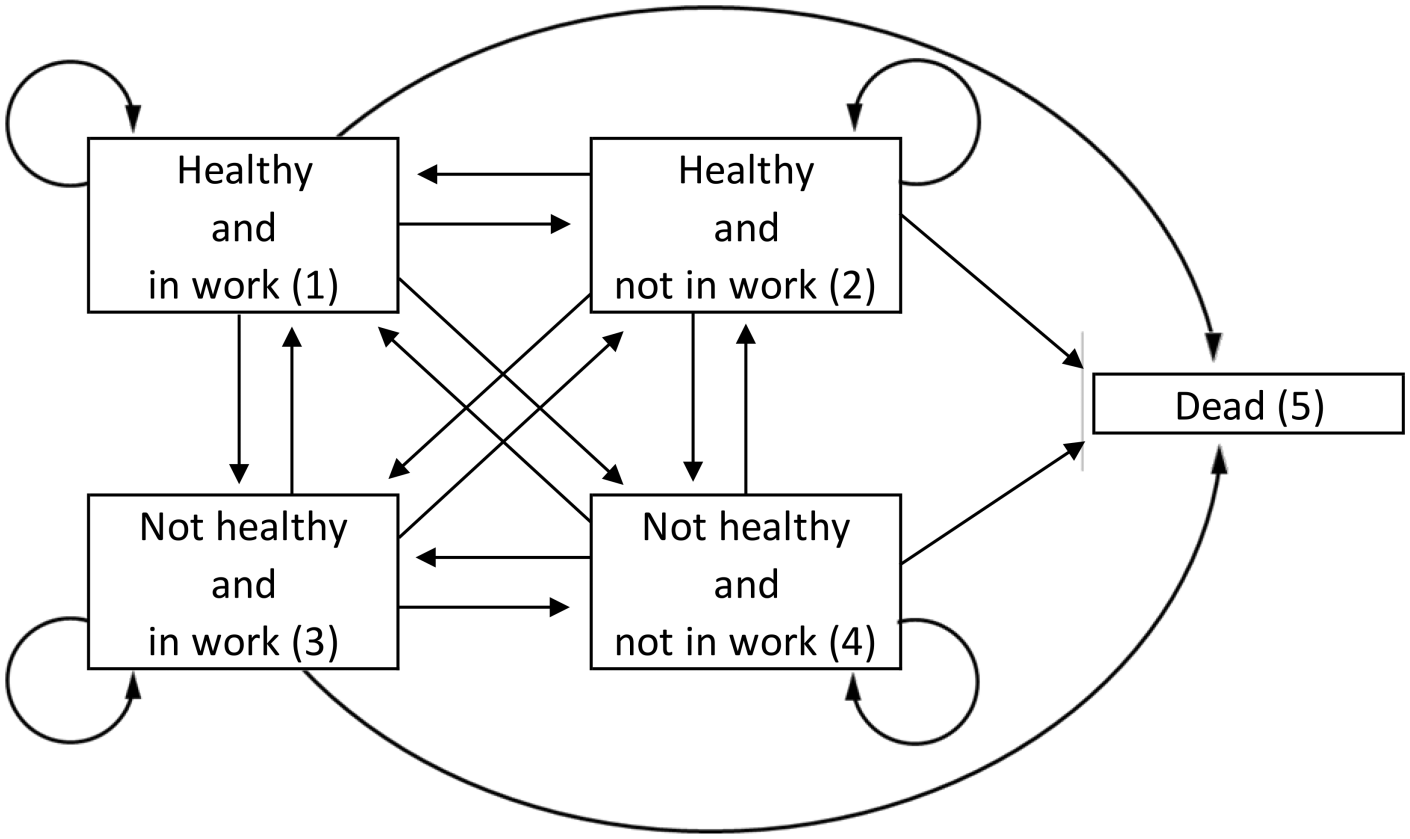
Multistate model of healthy working life expectancy: states and permitted transitions (shown with arrow heads).

“Work” was defined as currently participating in paid work if “working now” was indicated as the current employment item (“Are you working now, temporarily laid off, unemployed and looking for work, disabled and unable to work, retired, a homemaker, or what?”). At each survey wave, respondents were classified as dead or alive and in one of four health and working states (healthy and in work, healthy and not in work, not healthy and in work, and not healthy and not in work) (Figure 1).

### Arthritis

Arthritis status was identified at each time point from self‐report of ever having received a doctor diagnosis of arthritis or rheumatism. We assumed that development of arthritis was irreversible (ie, once reported, arthritis would continue to be present) and that arthritis would not have been present before a negative survey response.

### Covariates

Age was included in transition probability models and was calculated as the number of years from date of birth to date of observation (either interview date or death date). Sex was classed as male or female. Ethnicity was classed as Black or African American, White, or other. Education was operationalized as a binary high school education variable. Participants had a high school education if they reported having a high school diploma, General Educational Development certificate, or a higher‐level qualification. Based on the geographic classification of the US population, states were grouped into the four regions that are used by the US Census Bureau:

- Midwest: Illinois, Indiana, Iowa, Kansas, Michigan, Minnesota, Missouri, Nebraska, North Dakota, Ohio, South Dakota, and Wisconsin
- Northeast: Connecticut, Maine, Massachusetts, New Hampshire, New Jersey, New York, Pennsylvania, Rhode Island, and Vermont
- South: Alabama, Arkansas, Delaware, District of Columbia, Florida, Georgia, Kentucky, Louisiana, Maryland, Mississippi, North Carolina, Oklahoma, South Carolina, Tennessee, Texas, Virginia, and West Virginia
- West: Alaska, Arizona, California, Colorado, Hawaii, Idaho, Montana, Nevada, New Mexico, Oregon, Utah, Washington, and Wyoming

We included obesity, which is common in the United States, and can add to functional limitation of arthritis.^10^, ^11^ Obesity was classed as a BMI of 30 or higher using self‐reported height (“About how tall are you?”) and bodyweight (“About how much do you weigh?”) to calculate.

### Missing data handling

Missing month of birth was taken as month 6 (June). Missing months in death records were imputed as month 6 (June), except when this conflicted with a survey participation date, in which case month 12 (December) was imputed. Month of death was also moved to month 12 (December) in cases of conflicting survey participation and death months in which both occurred in the same year (in this case, recorded month of death was considered erroneous). Records of deaths without death dates were ignored.

We excluded participants with missing data in any of the fixed (time‐invariant) covariates (sex, ethnicity, address region, education). We also excluded participants for whom data were missing at all contributing time points in any of the time‐varying covariates (arthritis, obesity); if a time‐varying covariate had missing data but was observed at any wave, then we imputed missing values with the value at the nearest earlier or later wave.

### Statistical analysis

HWLE was estimated with interpolated Markov chain multistate modeling of cross‐longitudinal survey data (panel data from repeated cross‐sectional surveys of a cohort). A multistate model was defined. HWLE status for Interpolated Markov Chains (IMaCh) analysis was 1 (healthy and working), 2 (healthy and not working), 3 (not healthy and working), 4 (not healthy and not working), or −1 (if health and/or work data were missing). HWLE status for IMaCh analysis was −2 (unknown vital and health/work status) at waves that were not participated in because time‐varying covariate data were not collected and so contributions to the likelihood of survival (HWLE status −1) could not be used.

Interpolated Markov chain software (IMaCh version 0.99r) was used to estimate HWLE with SEs.^8^, ^9^ This approach uses multinomial logistic regression to model the probabilities of transition from and to each HWLE state (or transition from alive to dead vital status) over small discrete time intervals (interpolation steps; in this analysis, 12‐month intervals) based on the transitions observed in the data, in which the analyzed time intervals are typically briefer than the time between data time points. Maximum likelihood estimates of transition probability model parameters were found by evaluating the product of the transition probabilities for each step contained within each observed transition (health and working states at consecutive observed time points) or sequence of transitions (in which more than one transition was observed for an individual). HWLE was estimated according to the health and working state observed at age 50 years and averaged (weighted by the observed prevalence of being in each health and working state at age 50 years) across the study period to estimate HWLE for the population. Further details are provided elsewhere.^8^, ^9^

Health expectancies were also estimated for time spent healthy and not in work, time spent not healthy and in work, and time spent not healthy and not in work. Life expectancy was estimated as the sum of all four health expectancies.

HWLE was estimated overall for the United States and then by sex, education, and ethnicity and for each of the four regions. HWLE was then estimated for those with and without arthritis and then by sex, education, ethnicity, region, and obesity status. Ethnicity estimates were only produced for Black or African American and White participants due to the small sample size in the “other” ethnicity group.

### Sensitivity analyses

To investigate the sensitivity of HWLE estimates to the calendar period, providing the basis for the prevalence of a healthy and working state at age 50 years (the starting point for computing health expectancies using the observed transitions), the observed prevalence of occupying each health and working state was plotted for the main survey years throughout the study period (1994–2020) for participants aged 55, 60, and 65 years at those time points (the HRS refreshment schedule meant that not all survey waves included adults aged exactly 50 years). Trends over calendar time were assessed visually.

## RESULTS

### Sample characteristics

The study sample comprised 37,062 respondents (20,461 women and 16,601 men) across 14 waves (Table 1). Participant demographics for individual waves (1994, 2000, 2010, and 2020) are given in Supplementary Table S1.

**Table 1:** Observed and weighted numbers and percentages of variables (including HWLE state), appearing in any HRS wave*

	Unweighted		Weighted	
	Frequency	%	Frequency	%
Total participants	37,062	–	157,237,565	–
Sex				
Female	20,461	55.2	82,452,003	52.4
Male	16,601	44.8	74,785,562	47.6
Education				
Less than high school	8,705	23.5	27,341,053	17.4
High school education	28,357	76.5	129,896,512	82.6
Region				
Northeast	6,458	17.4	29,996,464	19.1
Midwest	8,331	22.5	38,010,716	24.2
South	16,438	44.4	63,036,362	40.1
West	5,835	15.7	26,194,023	16.7
Ethnicity				
Black	7,160	19.3	17,775,468	11.3
White	26,828	72.4	126,465,285	80.4
Other	3,074	8.3	12,996,812	8.3
Ever diagnosed with arthritis?				
No	12,061	32.5	58,039,716	36.9
Yes	25,001	67.5	99,197,849	63.1
Obese				
No	21,987	59.3	92,364,951	58.7
Yes	15,075	40.7	64,872,614	41.3

* HRS, Health and Retirement Survey; HWLE, healthy working life expectancy.

In the United States overall, life expectancy was 31.31 years (95% confidence interval [CI] 31.03–31.41) from age 50 years (men: 29.40 years [95% CI 29.12–29.68]; women: 32.82 years [95% CI 32.58–33.07]) and HWLE was 9.36 years (95% CI 9.26–9.46) from age 50 years (men: 10.03 years [95% CI 9.88–10.18]; women: 8.76 years [95% CI 8.63–8·88]). Supplementary Table S2 shows HWLE and life expectancy by sex, educational attainment, ethnic group, US region, and obesity.

### HWLE in adults with arthritis

HWLE for those with arthritis was 6.18 years (95% CI 6.06–6.30; men: 6.10 years [95% CI 5.91–6.30]; women: 6.05 years [95% CI 5.90–6.20]), approximately half that of those without arthritis, whose HWLE was 11.71 years (95% CI 11.56–11.86; men: 12.36 years [95% CI 12.14–12.58]; women: 11.12 years [95% CI 10.91–11.34]; Table 2, Supplementary Table S2). HWLE made up 22% of life expectancy from age 50 years for men with arthritis and 19% of life expectancy from age 50 years for women with arthritis (Table 2). The amount of time participants were healthy and not in work, not healthy and in work, and not healthy and not in work is presented in Supplementary Table S3 for men and Supplementary Table S4 for women.

**Table 2:** Estimates of HWLE overall and as a percentage of LE for adults aged 50 years and over who have arthritis, stratified by sex, education, region, ethnicity, and obesity*

	Male			Female		
	HWLE, years (95% CI)	LE, years (95% CI)	% HWLE of LE	HWLE, years (95% CI)	LE, years (95% CI)	% HWLE of LE
Overall						
No arthritis	12.36 (12.14–12.58)	30.18 (29.73–30.63)	41	11.12 (10.91–11.34)	33.44 (32.94–33.94)	33
Arthritis	6.10 (5.91–6.30)	27.91 (27.52–28.31)	22	6.05 (5.90–6.20)	32.00 (31.69–32.30)	19
Education						
Less than high school						
No arthritis	9.86 (9.38–10.35)	27.09 (26.25–27.92)	36	7.22 (6.72–7.71)	30.07 (29.10–31.04)	24
Arthritis	2.41 (2.09–2.72)	24.02 (23.21–24.83)	10	3.72 (3.45–3.99)	28.90 (28.35–29.45)	13
High school education						
No arthritis	12.70 (12.46–12.95)	30.89 (30.34–31.45)	41	11.54 (11.30–11.77)	34.14 (33.54–34.74)	34
Arthritis	6.75 (6.52–6.97)	28.91 (28.44–29.38)	23	6.43 (6.25–6.60)	32.86 (32.49–33.22)	20
Region						
Northeast						
No arthritis	13.18 (12.67–13.69)	31.62 (30.50–32.73)	42	11.40 (10.88–11.92)	33.25 (32.07–34.43)	34
Arthritis	6.27 (5.71–6.82)	28.64 (27.66–29.62)	22	6.79 (6.60–6.99)	32.28 (31.54–33.01)	21
Midwest						
No arthritis	11.17 (10.71–11.63)	29.71 (28.78–30.64)	38	11.46 (11.02–11.90)	34.27 (33.24–35.31)	33
Arthritis	5.07 (4.66–5.48)	27.62 (26.77–28.48)	18	6.90 (6.58–7.21)	31.74 (31.13–32.34)	22
West						
No arthritis	11.70 (11.15–12.25)	31.12 (30.08–32.17)	38	10.66 (10.12–11.20)	33.39 (32.21–34.56)	32
Arthritis	8.24 (7.66–8.81)	30.44 (29.51–31.38)	27	6.74 (6.16–7.33)	33.80 (33.09–34.52)	20
South						
No arthritis	12.74 (12.41–13.07)	29.27 (28.57–29.97)	44	10.95 (10.61–11.28)	32.98 (32.18–33.78)	33
Arthritis	6.39 (6.09–6.69)	26.85 (26.25–27.44)	24	6.25 (6.01–6.49)	31.48 (31.02–31.95)	20
Ethnicity						
Black or African American						
No arthritis	10.35 (9.85–10.85)	26.94 (25.75–28.13)	38	10.04 (9.55–10.53)	28.49 (27.28–29.70)	35
Arthritis	3.51 (3.12–3.89)	25.38 (24.38–26.38)	14	4.73 (4.43–5.03)	29.36 (28.60–30.13)	16
White						
No arthritis	12.81 (12.56–13.06)	30.59 (30.09–31.08)	42	11.29 (11.04–11.54)	34.02 (33.46–34.58)	33
Arthritis	6.88 (6.64–7.11)	28.46 (28.03–28.90)	24	6.29 (6.10–6.47)	32.47 (32.13–32.81)	19
Obesity						
Nonobese						
No arthritis	12.78 (12.54–13.01)	30.03 (29.56–30.5)	43	11.74 (11.47–12.00)	33.68 (33.17–34.18)	35
Arthritis	6.51 (6.27–6.74)	27.23 (26.77–27.69)	24	6.81 (6.61–7.01)	31.85 (31.50–32.21)	21
Obese						
No arthritis	11.50 (11.17–11.84)	31.29 (30.51–32.07)	37	10.07 (9.76–10.38)	33.36 (32.59–34.14)	30
Arthritis	5.48 (5.24–5.72)	29.82 (29.10–30.54)	18	5.12 (4.93–5.31)	32.88 (32.29–33.47)	16

* CI, confidence interval; HWLE, healthy working life expectancy; LE, life expectancy.

**Figure 2:**
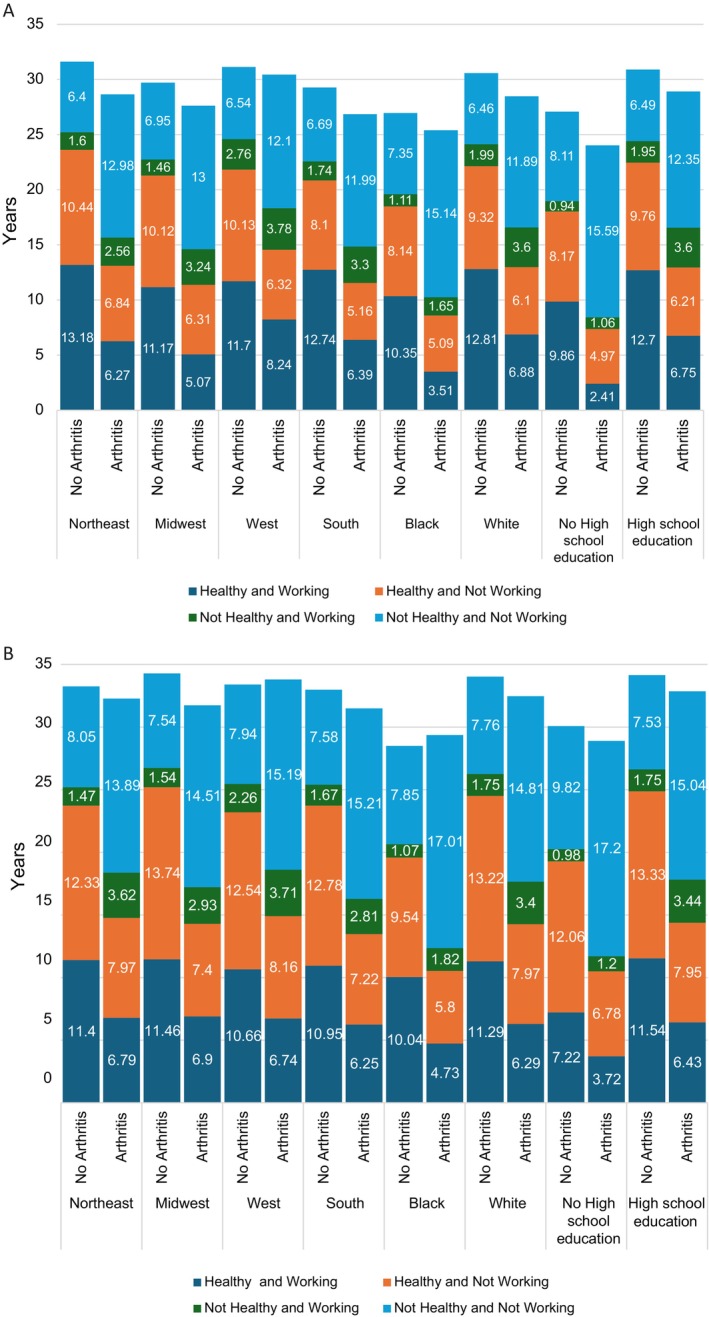
(A) Healthy working life expectancy for men with and without arthritis, stratified by region, ethnicity, and education level. (B) Healthy working life expectancy for women with and without arthritis, stratified by region, ethnicity, and education level.

Among those with arthritis, HWLE was higher for those with a high school education compared to those without a high school education (men: 6.75 years [95% CI 6.52–6.97] compared to 2.41 years [95% CI 2.09–2.72]; women: 6.43 years [95% CI 6.25–6.60] compared to 3.72 years [95% CI 3.45–3.99]; Table 2). HWLE was lower for the Black or African American group than the White group (Supplementary Table S2). Among those with arthritis, HWLE by ethnic group was lowest for Black or African American men (3.51 years [95% CI 3.12–3.89]), followed by Black or African American women (4.73 years [95% CI 4.43–5.03]) and White women (6.29 years [95% CI 6.10–6.47]), and was highest for White men (6.88 years [95% CI 6.64–7.11]) (Table 2). For men with arthritis, HWLE was lower in the Midwest region of the United States (5.07 years [95% CI 4.66–5.48]) and highest in the West (8.24 years [95% CI 7.66–8.81]) (Table 2, Figure 2A). For women with arthritis, there was less variation than for men, with the lowest HWLE in the South (6.25 years [95% CI 6.01–6.49]) and the highest HWLE in the Midwest (6.90 years [95% CI 6.58–7.21]) (Table 2, Figure 2B). HWLE for those with obesity but without arthritis was 11.50 years (95% CI 11.17–11.84) for men and 10.07 years (95% CI 9.76–10.38) for women, which was lower than the HWLE for those without obesity and without arthritis (men: 12.78 years [95% CI 12.54–13.01]; women: 11.74 years [95% CI 11.47–12.00]) but notably higher than the HWLE for those with arthritis and without obesity (men: 6.51 years [95% CI 6.27–6.74]; women: 6.81 years [95% CI 6.61–7.01]) (Table 2). HWLE was lowest for those with both arthritis and obesity: 5.48 years (95% CI 5.24–5.72) from age 50 years for men and 5.12 years (95% CI 4.93–5.31) from age 50 years for women (Table 2). Estimates for obesity were produced overall and for covariates (see Supplementary Tables S5 and S6).

### Sensitivity analysis

For each wave of the data, the prevalence rate of a healthy and working state was calculated at age 50, 60, and 65 years to look at whether the results would be sensitive to the years used to estimate the prevalence. At all ages, the prevalence within the data was within similar range, indicating that the calendar period selected may not be sensitive to the years included and therefore the last 10 years chosen.

## DISCUSSION

This study used data from a large nationally representative longitudinal study to estimate the average amount of time that adults who have arthritis in the United States are healthy and in work from age 50 years. Arthritis is associated with a significant impact on healthy working life; HWLE is reduced by almost half in people with arthritis compared to those who do not have arthritis, an indicator of the significant extent of the role that joint pain and related disability have on being healthy and in work. Assuming a 260‐day working year, on average, people with arthritis are healthy and in work for approximately 1,400 fewer days than people without arthritis. Across the United States, approximately 20 million people aged between 50 and 64 years have arthritis, which suggests around 28 billion days are lost across working life in this age group.

HWLE is higher for men than women overall but is similar for men and women with arthritis. It is particularly low—fewer than 4 years from age 50 years—among those with less than a high school education and among Black or African American men. In these groups, differences in life expectancy associated with arthritis were smaller than differences in healthy working years, meaning HWLE also made up a much smaller portion of life expectancy from age 50 years compared to other groups.

The relationship with education reflects the direct impact of education itself but also acts as a proxy for socioeconomic status and occupation (eg, low education attainment is associated with manual laborers). Lower educational attainment means people have less capacity to adapt or change roles and there is less opportunity to manage the impacts of joint pain and disability on employment.^14^ Often, the occupations associated with low educational attainment have less latitude to offer accommodations for pain and disability, resulting in people leaving employment. Differences by region were expected in line with variation with other factors, such as levels of disability and unemployment.^15^, ^16^ Variation by region and sex (by region) can be explained by differences in job opportunities and available occupations in regions. For example, in the Midwest, the reduction in the manufacturing industries and an increase in health care will partially explain the lower HWLE in men and higher HWLE in women.

Notably, although people with obesity but without arthritis have lower HWLE than those without obesity or arthritis, their HWLE remains above the national average (11.5 years vs 9.4 years). In contrast, people with arthritis have substantially lower HWLE than the national average. This can be partially explained by the longer natural history of obesity and with it being present for a longer period, during which time people will have adapted and may have changed their occupation to accommodate its impact on work capacity (eg, by moving to sedentary jobs with low physical demand).^17^

The time of onset of pain and disability may also help explain the greater reduction in HWLE for people with arthritis, as the impact condition increases from age 50 years onward and thus has a greater relative effect in those aged >50 years. Having increasing symptoms beginning at this later age results in less opportunity for adaptation at work and helps explain the relationship with educational attainment.

The variation by health and sociodemographic factors indicates that there is potential for HWLE to increase. The other health and working states also indicate that other issues could contribute to extending working lives. The amount of time in which adults with arthritis (5.9 and 7.6 years for men and women with arthritis, respectively) are healthy but not in work suggests that more employment opportunities would allow more adults to extend their working lives. Some of this time can also be attributable to adults who have chosen to retire because they have the financial means. Notably, people in this position may choose to leave work because work is no longer fulfilling or rewarding, which highlights the role of job opportunities and accommodations for increasing HWLE. The extent of time that adults are not healthy and in work (3.2 and 3.1 years for men and women with arthritis, respectively) indicates the amount of time that may be spent with absenteeism and presenteeism, receiving accommodations to remain in work (eg, flexible working to manage pain or, for those with lower limb pain, support to reduce weight‐bearing), or in occupations in which their arthritis does not have an impact on work ability.

The overall HWLE (10.4 years) and differences by sex and education are similar to those for England.^8^ The point estimate for HWLE for those with arthritis (6.1 years [95% CI 5.91–6.30]) was slightly higher in the United States than that for those with osteoarthritis in England (5.68 years [95% CI 5.29–6.07]).^9^ Similar to this study, differences by geography occur in England.^8^, ^9^

The strengths of this study include the use of a large sample size from 14 survey time points from a nationally representative dataset. The life expectancy estimated in this project is similar to those produced by the United States Social Security Administration (https://www.ssa.gov/oact/STATS/table4c6.html). The definition of health includes the capacity to work and aligns with the recommended Global Activity Limitation Indicator (GALI) and allows comparison with other HWLE indicators. The definition of work is broad, and one limitation is that it does not differentiate between amounts of work (eg, full‐time vs part‐time); although reducing hours is one way for people with arthritis to stay in employment, this population measure does not account for amount of time worked. The need for a binary categorization of work prevents estimation of time in important working states such as temporarily laid off, unemployed and looking for work, disabled and unable to work, retired, or moving out of employment into a caring role. Although there is an association between education and occupation, including the physical demands of work, some of the nuanced HWLE will differ within each education level or by occupation. Reasons for not working can be due to people being able to choose to leave work because they are financially able as well as because there are limited job opportunities that meet their work capacity. For the health definition, participants were only asked about the duration of the health problem (either temporary or lasting three months or longer) if they reported limitation in the first of the series of three questions (limited in the kind or amount of paid work). When activity limitation was identified in subsequent questions, either because a participant did not have a work limitation or because they felt the question was not applicable, it was not possible to determine the temporariness, and therefore it was not possible to incorporate the temporality aspect of the recommended GALI.^18^ The use of panel data collected every two years will miss some transitions and overestimate HWLE for people with arthritis if there are additional periods when they are not healthy or in work.

Self‐report of doctor‐diagnosed arthritis excludes those who have joint pain but do not seek health care for it, perhaps more so for those with osteoarthritis who are less likely to seek health care as symptoms can be viewed as an inevitable part of aging and that health care will not impact this. However, this question does ensure that most arthritis that leads to significant functional limitation is captured, as most people would seek health care advice for it.^19^ Incorrect recall of diagnosis may lead to misclassification of arthritis and an overestimate of HWLE for people with arthritis. The binary variable masks the heterogeneity in terms of type, severity, and time since diagnosis of arthritis; HRS data prevent further stratification by arthritis. Analysis of HWLE by different types of arthritis is not possible due to a lack of information and sample size. The sensitivity analysis indicates that there was no impact when people were diagnosed with arthritis (ie, no period effects). Self‐reported height and weight can lead to misclassification and lower estimates of obesity in populations^20^; obesity levels in this study were similar (40.7%) compared to the US estimate of 40%. The sample size prevents further stratification of, for example, region and ethnicity by education; this would provide greater information on targets to increase healthy working lives. The binary classification of education prevents estimates of HWLE linked to levels associated with occupation. The impact of exclusion of those with missing data is unclear; it may have led to an under or overestimate of HWLE.

With population aging, people choosing to work until they are older, and increasing risk factors (such as obesity) in the United States, the prevalence of arthritis among workers aged 50 years and over will increase. The lower HWLE in adults with arthritis suggests that extending the length of work participation will be difficult for many people who experience pain and functional limitation from arthritis due to the range of joint conditions. However, having arthritis, or indeed obesity, does not mean that functional limitation is inevitable. Differences in HWLE by sex (by region), socioeconomic status, and geographic region identifies subpopulations who are target groups for interventions either to improve health or work opportunities and conditions (including initiatives to promote job availability and fair hiring practices, facilitate training and retraining opportunities for older workers, and encourage employer flexibility to adapt jobs and workplaces). Research is required to understand—not just in those with arthritis and obesity but also in the general population—how health, sociodemographic, workplace, and environmental factors combine to drive being healthy and in work; this includes understanding why absenteeism (work absence, eg, for health reasons) and presenteeism (reduced productivity while at work due to illness or for other reasons) occur, which is picked up in the other health and working states in this study. Focusing on different occupations and other life‐course demands would provide a basis for this but requires datasets that are sufficiently large enough to allow modeling of transitions and include content across health and workplace factors. Approaches to self‐management of arthritis, in terms of how to manage symptoms and maintain functional capacity, will help to change perceptions that disability is a likely consequence of arthritis. Further investigation is needed to determine the extent that interference of symptomatic arthritis with work is reduced by supportive workplaces (including accommodations and flexibility) and a higher degree of individual control of work responsibilities and arrangements. Proactive population and preventive medicine approaches targeting maintenance of workers’ health to prevent arthritis and obesity impacting work participation could have a significant impact on the ability of populations to extend working lives. Early identification (perhaps via presenteeism) and interventions could extend healthy working lives not just for those with arthritis but for all workers.

In conclusion, the extent of the reduction of time in a healthy and working state highlights the societal impact of arthritis. Variation in the amount of time in a healthy and working state by sociodemographic factors suggests that there are approaches that can increase HWLE. Further exploration of workplace factors and employment opportunities is required to identify how to reduce the impact of arthritis on healthy working lives.

## AUTHOR CONTRIBUTIONS

All authors contributed to at least one of the following manuscript preparation roles: conceptualization AND/OR methodology, software, investigation, formal analysis, data curation, visualization, and validation AND drafting or reviewing/editing the final draft. As corresponding author, Professor Wilkie confirms that all authors have provided the final approval of the version to be published, and takes responsibility for the affirmations regarding article submission (eg, not under consideration by another journal), the integrity of the data presented, and the statements regarding compliance with institutional review board/Declaration of Helsinki requirements.

## Supporting information


**Table S1:** Observed and weighted numbers and percentages of variables (including HWLE state occupation), at HRS waves 1994, 2000, 2010, and 2020
**Table S2:** Life Expectancy and Healthy Working life Expectancy for Overall US population aged 50, and stratified by sex, education, region, ethnicity
**Table S3:** All health and work life expectancies for males with and without arthritis
**Table S4:** All health and work life expectancies for females with and without arthritis
**Table S5:** All health and work life expectancies for males with and without obesity
**Table S6:** All health and work life expectancies for females with and without obesity

## References

[1] National Academies of Sciences, Engineering, and Medicine; Division of Behavioral and Social Sciences and Education; Committee on National Statistics; Committee on Population; Committee on Understanding the Aging Workforce and Employment at Older Ages; Becker T , Fiske ST , eds. Understanding the Aging Workforce: Defining a Research Agenda. National Academies Press; 2022. https://www.ncbi.nlm.nih.gov/books/NBK588545/ 36630548

[2] Fallon EA , Boring MA , Foster AL , et al. Prevalence of diagnosed arthritis ‐ United States, 2019‐2021. MMWR Morb Mortal Wkly Rep 2023;72(41):1101–1107. 37824422 10.15585/mmwr.mm7241a1 PMC10578950

[3] Hootman JM , Helmick CG , Barbour KE , et al. Updated projected prevalence of self‐reported doctor‐diagnosed arthritis and arthritis‐attributable activity limitation among US adults, 2015–2040. Arthritis Rheumatol 2016;68(7):1582–1587. 27015600 10.1002/art.39692 PMC6059375

[4] Waddell G , Kim Burton A . Is Work Good for Your Health and Well‐being? Springer; 2006.

[5] Theis KA , Murphy LB , Guglielmo D , et al. Prevalence of arthritis and arthritis‐attributable activity limitation ‐ United States, 2016‐2018. MMWR Morb Mortal Wkly Rep 2021;70(40):1401–1407. 34618800 10.15585/mmwr.mm7040a2 PMC8519273

[6] Murphy LB , Cisternas MG , Pasta DJ , et al. Medical expenditures and earnings losses among US adults with arthritis in 2013. Arthritis Care Res (Hoboken) 2018;70(6):869–876. 28950426 10.1002/acr.23425

[7] Lièvre A , Jusot F , Barnay T , et al. Healthy working life expectancies at age 50 in Europe: a new indicator. J Nutr Health Aging 2007;11(6):508–514. 17985068

[8] Parker M , Bucknall M , Jagger C , Wilkie R . Population‐based estimates of healthy working life expectancy in England at age 50 years: analysis of data from the English Longitudinal Study of Ageing. Lancet Public Health 2020;5(7):e395–e403. 32619541 10.1016/S2468-2667(20)30114-6

[9] Lynch M , Bucknall M , Jagger C , et al. Demographic, health, physical activity, and workplace factors are associated with lower healthy working life expectancy and life expectancy at age 50. Sci Rep 2024;14(1):5936. 38467680 10.1038/s41598-024-53095-z PMC10928117

[10] Nedunchezhiyan U , Varughese I , Sun AR , et al. Obesity, inflammation, and immune system in osteoarthritis. Front Immunol 2022;13:907750. 35860250 10.3389/fimmu.2022.907750 PMC9289681

[11] Dall TM , Sapra T , Natale Z , et al. Assessing the economic impact of obesity and overweight on employers: identifying opportunities to improve work force health and well‐being. Nutr Diabetes 2024;14(1):96. 39632835 10.1038/s41387-024-00352-9 PMC11618327

[12] Emmerich SD , Fryar CD , Stierman B , et al. Obesity and severe obesity prevalence in adults: United States, August 2021–August 2023. NCHS Data Brief 2024;(508). doi:10.15620/cdc/159281 PMC11744423 39808758

[13] Kudel I , Huang JC , Ganguly R . Impact of obesity on work productivity in different US occupations: analysis of the National Health and Wellness Survey 2014 to 2015. J Occup Environ Med 2018;60(1):6–11. 29065062 10.1097/JOM.0000000000001144 PMC5770108

[14] Lynch M , Wilkie R . Increasing inequalities in healthy working lives. Lancet Public Health 2023;8(8):e578–e579. 37516473 10.1016/S2468-2667(23)00155-X

[15] Crankshaw K . The South had highest disability rate among regions in 2021. US Census Bureau. 2023. Accessed January 7, 2025. https://www.census.gov/library/stories/2023/06/disability-rates-higher-in-rural-areas-than-urban-areas.html#:~:text=Regional%20Differences%20in%20Disability,for%20a%20variety%20of%20reasons

[16] Yu W. What explains the variation in unemployment rates across the United States? 2022. Accessed January 7, 2025. https://anderson-review.ucla.edu/wp-content/uploads/2022/11/Yu_2209_report04.pdf

[17] Canizares M , Hogg‐Johnson S , Gignac MAM , et al. Increasing trajectories of multimorbidity over time: birth cohort differences and the role of changes in obesity and income. J Gerontol B Psychol Sci Soc Sci 2018;73(7):1303–1314. 28199711 10.1093/geronb/gbx004

[18] Robine JM , Jagger C ; Euro‐REVES Group . Creating a coherent set of indicators to monitor health across Europe: the Euro‐REVES 2 project. Eur J Public Health 2003;13(3 suppl):6–14. 14533742 10.1093/eurpub/13.suppl_1.6

[19] Paskins Z , Sanders T , Hassell AB . What influences patients with osteoarthritis to consult their GP about their symptoms? A narrative review. BMC Fam Pract 2013;14(1):195. 24359101 10.1186/1471-2296-14-195 PMC3890599

[20] Hodge JM , Shah R , McCullough ML , et al. Validation of self‐reported height and weight in a large, nationwide cohort of U.S. adults. PLoS One 2020;15(4):e0231229. 32282816 10.1371/journal.pone.0231229 PMC7153869

