# Supplementary material for: How Long Do People With Arthritis Stay Healthy and in Work? Analysis of Data From the Health and Retirement Study
Source: ACR Open Rheumatol. 2026 Jan 20;8(1):e70142. doi: 10.1002/acr2.70142 (PMC12820344; doi:10.1002/acr2.70142)
Supplement: Supplementary file 1 — Table S1: Observed and weighted numbers and percentages of variables (including HWLE state occupation), at HRS waves 1994, 2000, 2010, and 2020 Table S2: Life Expectancy and Healthy Working life Expectancy for Overall US population aged 50, and stratified by sex, education, region, ethnicity Table S3: All health and work life expectancies for males with and without arthritis Table S4: All health and work life expectancies for females with and without arthritis Table S5: All health and work life expectancies for males with and without obesity Table S6: All health and work life expectancies for females with and without obesity [file ACR2-8-e70142-s001.docx]

**Supplementary Data**

**Table S1: Observed and weighted numbers and percentages of variables (including HWLE state occupation), at HRS waves 1994, 2000, 2010, and 2020**

|  | 1994 | | 1994 | | 2000 | | 2000 | | 2010 | | 2010 | | 2020 | | 2020 | |
| --- | --- | --- | --- | --- | --- | --- | --- | --- | --- | --- | --- | --- | --- | --- | --- | --- |
|  | Unweighted | | Weighted | | Unweighted | | Weighted | | Unweighted | | Weighted | | Unweighted | | Weighted | |
|  | Freq | % | Freq | % | Freq | % | Freq | % | Freq | % | Freq | % | Freq | % | Freq | % |
| Total Participants | 10,307 |  | 27,655,018 |  | 18,791 |  | 65,123,638 |  | 20,868 |  | 91,588,899 |  | 14,785 |  | 73,638,730 |  |
| Sex |  |  |  |  |  |  |  |  |  |  |  |  |  |  |  |  |
| Female | 5,372 | 52.1% | 14,070,170 | 50.9% | 10,822 | 57.6% | 36,673,475 | 56.3% | 11,940 | 57.2% | 49,055,812 | 53.6% | 8,628 | 58.4% | 39,712,651 | 53.9% |
| Male | 4,935 | 47.9% | 13,584,848 | 49.1% | 7,969 | 42.4% | 28,450,163 | 43.7% | 8,928 | 42.8% | 42,533,087 | 46.4% | 6,157 | 41.6% | 33,926,079 | 46.1% |
| Education |  |  |  |  |  |  |  |  |  |  |  |  |  |  |  |  |
| Less than high school | 2,722 | 26.4% | 6,277,750 | 22.7% | 5,165 | 27.5% | 15,216,891 | 23.4% | 4,104 | 19.7% | 13,458,499 | 14.7% | 2,242 | 15.2% | 7,764,106 | 10.5% |
| High school education | 7,585 | 73.6% | 21,377,268 | 77.3% | 13,626 | 72.5% | 49,906,747 | 76.6% | 16,764 | 80.3% | 78,130,400 | 85.3% | 12,543 | 84.8% | 65,874,624 | 89.5% |
| Region |  |  |  |  |  |  |  |  |  |  |  |  |  |  |  |  |
| North East | 1,832 | 17.8% | 5,928,676 | 21.4% | 3,293 | 17.5% | 12,324,849 | 18.9% | 3,731 | 17.9% | 17,583,101 | 19.2% | 2,481 | 16.8% | 13,742,072 | 18.7% |
| Midwest | 2,515 | 24.4% | 6,756,001 | 24.4% | 4,703 | 25.0% | 16,742,401 | 25.7% | 4,857 | 23.3% | 22,664,665 | 24.7% | 3,145 | 21.3% | 17,725,101 | 24.1% |
| South | 4,327 | 42.0% | 9,586,780 | 34.7% | 7,572 | 40.3% | 23,705,387 | 36.4% | 9,259 | 44.4% | 37,500,320 | 40.9% | 6,833 | 46.2% | 30,043,692 | 40.8% |
| West | 1,633 | 15.8% | 5,383,561 | 19.5% | 3,223 | 17.2% | 12,351,001 | 19.0% | 3,021 | 14.5% | 13,840,813 | 15.1% | 2,326 | 15.7% | 12,127,865 | 16.5% |
| Ethnicity |  |  |  |  |  |  |  |  |  |  |  |  |  |  |  |  |
| Black | 1,683 | 16.3% | 2,543,994 | 9.2% | 2,600 | 13.8% | 5,772,772 | 8.9% | 4,020 | 19.3% | 10,394,199 | 11.3% | 3,260 | 22.0% | 8,584,084 | 11.7% |
| White | 8,259 | 80.1% | 24,198,466 | 87.5% | 15,529 | 82.6% | 57,035,158 | 87.6% | 15,223 | 72.9% | 74,799,689 | 81.7% | 9,814 | 66.4% | 57,342,788 | 77.9% |
| Other | 365 | 3.5% | 912,558 | 3.3% | 662 | 3.5% | 2,315,708 | 3.6% | 1,625 | 7.8% | 6,395,011 | 7.0% | 1,711 | 11.6% | 7,711,858 | 10.5% |
| Diagnosed with Arthritis |  |  |  |  |  |  |  |  |  |  |  |  |  |  |  |  |
| No | 5,552 | 53.9% | 15,301,122 | 55.3% | 7,357 | 39.2% | 28,186,761 | 43.3% | 7,996 | 38.3% | 39,834,937 | 43.5% | 4,996 | 33.8% | 27,999,797 | 38.0% |
| Yes | 4,755 | 46.1% | 12,353,896 | 44.7% | 11,434 | 60.8% | 36,936,877 | 56.7% | 12,872 | 61.7% | 51,753,962 | 56.5% | 9,789 | 66.2% | 45,638,933 | 62.0% |
| Obese participant |  |  |  |  |  |  |  |  |  |  |  |  |  |  |  |  |
| No | 7,840 | 76.1% | 21,423,764 | 77.5% | 14,266 | 75.9% | 49,397,680 | 75.9% | 14,073 | 67.4% | 61,234,133 | 66.9% | 9,687 | 65.5% | 48,568,404 | 66.0% |
| Yes | 2,467 | 23.9% | 6,231,254 | 22.5% | 4,525 | 24.1% | 15,725,958 | 24.1% | 6,795 | 32.6% | 30,354,766 | 33.1% | 5,098 | 34.5% | 25,070,326 | 34.0% |
| HWLE |  |  |  |  |  |  |  |  |  |  |  |  |  |  |  |  |
| Unknown Status | 16 | 0.2% | 39,208 | 0.1% | 50 | 0.3% | 147,706 | 0.2% | 416 | 2.0% | 1,774,758 | 1.9% | 174 | 1.2% | 679,114 | 0.9% |
| Healthy and Working | 4885 | 47.4% | 13,321,037 | 48.2% | 5064 | 26.9% | 20,938,190 | 32.2% | 5848 | 28.0% | 31,913,534 | 34.8% | 3442 | 23.3% | 20,739,979 | 28.2% |
| Healthy and Not Working | 2151 | 20.9% | 5,850,436 | 21.2% | 6412 | 34.1% | 20,159,086 | 31.0% | 5533 | 26.5% | 21,174,075 | 23.1% | 4241 | 28.7% | 20,483,624 | 27.8% |
| Not Healthy and Working | 1041 | 10.1% | 2,892,953 | 10.5% | 1130 | 6.0% | 4,558,266 | 7.0% | 1534 | 7.4% | 8,440,951 | 9.2% | 919 | 6.2% | 5,710,772 | 7.8% |
| Not Healthy and Not Working | 2214 | 21.5% | 5,551,384 | 20.1% | 6135 | 32.6% | 19,320,390 | 29.7% | 7537 | 36.1% | 28,285,581 | 30.9% | 6009 | 40.6% | 26,025,241 | 35.3% |

**Table S2: Life Expectancy and Healthy Working life Expectancy for Overall US population aged 50, and stratified by sex, education, region, ethnicity**

|  | Life expectancy, years  (95% CI) | | | Healthy working life  expectancy, years  (95% CI) | | | Healthy & not working life expectancy (95% CI) | | | Not healthy and & working life expectancy (95% CI) | | | Not healthy & not working life expectancy (95% CI) | | | Healthy life expectancy | Working life expectancy |
| --- | --- | --- | --- | --- | --- | --- | --- | --- | --- | --- | --- | --- | --- | --- | --- | --- | --- |
| Overall | 31.22 | 31.03 | 31.41 | 9.36 | 9.26 | 9.46 | 8.40 | 8.28 | 8.51 | 2.41 | 2.36 | 2.46 | 11.06 | 10.92 | 11.20 | 17.76 | 11.77 |
| Sex |  |  |  |  |  |  |  |  |  |  |  |  |  |  |  |  |  |
| Male | 29.40 | 29.12 | 29.68 | 10.03 | 9.88 | 10.18 | 7.41 | 7.24 | 7.58 | 2.44 | 2.37 | 2.52 | 9.51 | 9.32 | 9.70 | 17.44 | 12.47 |
| Female | 32.82 | 32.58 | 33.07 | 8.76 | 8.63 | 8.88 | 9.20 | 9.03 | 9.36 | 2.39 | 2.32 | 2.46 | 12.48 | 12.28 | 12.67 | 17.96 | 11.15 |
| Education |  |  |  |  |  |  |  |  |  |  |  |  |  |  |  |  |  |
| High school educated | 32.04 | 31.81 | 32.26 | 9.79 | 9.68 | 9.90 | 7.51 | 7.27 | 7.74 | 1.17 | 1.09 | 1.25 | 13.13 | 12.83 | 13.43 | 17.30 | 10.96 |
| Not high school educated | 27.83 | 27.45 | 28.21 | 6.02 | 5.81 | 6.23 | 8.73 | 8.58 | 8.87 | 2.59 | 2.53 | 2.65 | 10.93 | 10.76 | 11.09 | 14.75 | 8.61 |
| Region |  |  |  |  |  |  |  |  |  |  |  |  |  |  |  |  |  |
| North East | 31.64 | 31.18 | 32.10 | 9.23 | 8.99 | 9.47 | 8.88 | 8.57 | 9.19 | 2.37 | 2.25 | 2.49 | 11.15 | 10.81 | 11.49 | 18.11 | 11.60 |
| Midwest | 31.13 | 30.74 | 31.51 | 9.32 | 9.12 | 9.51 | 8.68 | 8.43 | 8.92 | 2.26 | 2.16 | 2.36 | 10.87 | 10.59 | 11.15 | 18.00 | 11.58 |
| South | 30.48 | 30.19 | 30.76 | 9.5 | 9.35 | 9.65 | 7.72 | 7.55 | 7.90 | 2.31 | 2.24 | 2.39 | 10.94 | 10.73 | 11.15 | 17.22 | 11.81 |
| West | 32.58 | 32.13 | 33.03 | 9.18 | 8.93 | 9.43 | 9.08 | 8.78 | 9.38 | 2.98 | 2.84 | 3.12 | 11.34 | 11.00 | 11.68 | 18.26 | 12.16 |
| Ethnicity |  |  |  |  |  |  |  |  |  |  |  |  |  |  |  |  |  |
| Black or African American | 28.21 | 27.73 | 28.69 | 7.31 | 7.10 | 7.52 | 6.74 | 6.48 | 7.00 | 1.44 | 1.35 | 1.53 | 12.71 | 12.36 | 13.07 | 14.05 | 8.75 |
| White | 31.65 | 31.44 | 31.86 | 9.69 | 9.58 | 9.81 | 8.57 | 8.44 | 8.71 | 2.60 | 2.54 | 2.66 | 10.78 | 10.63 | 10.93 | 18.26 | 12.29 |
| Arthritis |  |  |  |  |  |  |  |  |  |  |  |  |  |  |  |  |  |
| No | 31.55 | 31.22 | 31.89 | 11.71 | 11.56 | 11.86 | 10.95 | 10.69 | 11.20 | 1.74 | 1.68 | 1.80 | 7.16 | 6.96 | 7.35 | 22.66 | 13.45 |
| Yes | 30.32 | 30.08 | 30.56 | 6.18 | 6.06 | 6.30 | 6.99 | 6.85 | 7.12 | 3.16 | 3.08 | 3.24 | 13.99 | 13.80 | 14.19 | 13.17 | 9.34 |
| Obesity |  |  |  |  |  |  |  |  |  |  |  |  |  |  |  |  |  |
| No | 31.06 | 30.85 | 31.28 | 10.14 | 10.02 | 10.26 | 9.05 | 8.90 | 9.19 | 2.09 | 2.04 | 2.15 | 9.79 | 9.64 | 9.93 | 19.19 | 12.23 |
| Yes | 32.36 | 31.93 | 32.79 | 8.00 | 7.84 | 8.15 | 6.81 | 6.60 | 7.01 | 2.99 | 2.90 | 3.09 | 14.56 | 14.21 | 14.92 | 14.81 | 10.99 |

**Table S3: All health and work life expectancies for males with and without arthritis**

|  |  |  |  |  |  |  |  |  |  |  |  |  |  |  |  |  |  |  |
| --- | --- | --- | --- | --- | --- | --- | --- | --- | --- | --- | --- | --- | --- | --- | --- | --- | --- | --- |
|  |  | Life expectancy, years | | | Healthy working life | | | Healthy & not working life expectancy (95% CI) | | | Not healthy and & working life expectancy (95% CI) | | | Not healthy & not working life expectancy (95% CI) | | | Healthy life expectancy | Working life expectancy |
|  |  | (95% CI) | | | expectancy, years | | |  |  |  |  |  |  |  |  |  |  |  |
|  |  |  | | | (95% CI) | | |  |  |  |  |  |  |  |  |  |  |  |
| **Overall** | No Arthritis | 30.18 | 29.73 | 30.63 | 12.36 | 12.14 | 12.58 | 9.34 | 9.01 | 9.67 | 1.83 | 1.74 | 1.91 | 6.65 | 6.40 | 6.90 | 21.70 | 14.19 |
|  | Arthritis | 27.91 | 27.52 | 28.31 | 6.10 | 5.91 | 6.30 | 5.92 | 5.71 | 6.12 | 3.19 | 3.06 | 3.32 | 12.7 | 12.40 | 13.00 | 12.02 | 9.29 |
| **Education** |  |  |  |  |  |  |  |  |  |  |  |  |  |  |  |  |  |  |
| Less than | No Arthritis | 27.09 | 26.25 | 27.92 | 9.86 | 9.38 | 10.35 | 8.17 | 7.61 | 8.72 | 0.94 | 0.80 | 1.08 | 8.11 | 7.6 | 8.63 | 18.03 | 10.8 |
| high school | Arthritis | 24.02 | 23.21 | 24.83 | 2.41 | 2.09 | 2.72 | 4.97 | 4.58 | 5.35 | 1.06 | 0.88 | 1.24 | 15.59 | 14.94 | 16.24 | 7.38 | 3.47 |
| High school education | No Arthritis | 30.89 | 30.34 | 31.45 | 12.7 | 12.46 | 12.95 | 9.76 | 9.34 | 10.17 | 1.95 | 1.85 | 2.05 | 6.49 | 6.19 | 6.78 | 22.46 | 14.65 |
|  | Arthritis | 28.91 | 28.44 | 29.38 | 6.75 | 6.52 | 6.97 | 6.21 | 5.96 | 6.46 | 3.6 | 3.44 | 3.76 | 12.35 | 12.01 | 12.69 | 12.96 | 10.35 |
| **Region** |  |  |  |  |  |  |  |  |  |  |  |  |  |  |  |  |  |  |
| North East | No Arthritis | 31.62 | 30.5 | 32.73 | 13.18 | 12.67 | 13.69 | 10.44 | 9.58 | 11.29 | 1.60 | 1.39 | 1.81 | 6.40 | 5.80 | 7.00 | 23.62 | 14.78 |
|  | Arthritis | 28.64 | 27.66 | 29.62 | 6.27 | 5.71 | 6.82 | 6.84 | 6.28 | 7.39 | 2.56 | 2.24 | 2.87 | 12.98 | 12.23 | 13.74 | 13.11 | 8.83 |
| Midwest | No Arthritis | 29.71 | 28.78 | 30.64 | 11.17 | 10.71 | 11.63 | 10.12 | 9.43 | 10.82 | 1.46 | 1.31 | 1.62 | 6.95 | 6.42 | 7.49 | 21.29 | 12.63 |
|  | Arthritis | 27.62 | 26.77 | 28.48 | 5.07 | 4.66 | 5.48 | 6.31 | 5.86 | 6.75 | 3.24 | 2.95 | 3.53 | 13.00 | 12.36 | 13.64 | 11.38 | 8.31 |
| West | No Arthritis | 31.12 | 30.08 | 32.17 | 11.7 | 11.15 | 12.25 | 10.13 | 9.32 | 10.93 | 2.76 | 2.49 | 3.03 | 6.54 | 5.95 | 7.12 | 21.83 | 14.46 |
|  | Arthritis | 30.44 | 29.51 | 31.38 | 8.24 | 7.66 | 8.81 | 6.32 | 5.81 | 6.83 | 3.78 | 3.38 | 4.19 | 12.1 | 11.37 | 12.83 | 14.56 | 12.02 |
| South | No Arthritis | 29.27 | 28.57 | 29.97 | 12.74 | 12.41 | 13.07 | 8.10 | 7.62 | 8.58 | 1.74 | 1.61 | 1.87 | 6.69 | 6.3 | 7.08 | 20.84 | 14.48 |
|  | Arthritis | 26.85 | 26.25 | 27.44 | 6.39 | 6.09 | 6.69 | 5.16 | 4.87 | 5.45 | 3.30 | 3.10 | 3.5 | 11.99 | 11.56 | 12.43 | 11.55 | 9.69 |
| **Ethnicity** |  |  |  |  |  |  |  |  |  |  |  |  |  |  |  |  |  |  |
| Black or African American | No Arthritis | 26.94 | 25.75 | 28.13 | 10.35 | 9.85 | 10.85 | 8.14 | 7.31 | 8.96 | 1.11 | 0.94 | 1.27 | 7.35 | 6.70 | 7.99 | 18.49 | 11.46 |
|  | Arthritis | 25.38 | 24.38 | 26.38 | 3.51 | 3.12 | 3.89 | 5.09 | 4.61 | 5.57 | 1.65 | 1.41 | 1.88 | 15.14 | 14.36 | 15.91 | 8.60 | 5.16 |
| White | No Arthritis | 30.59 | 30.09 | 31.08 | 12.81 | 12.56 | 13.06 | 9.32 | 8.95 | 9.68 | 1.99 | 1.89 | 2.1 | 6.46 | 6.18 | 6.74 | 22.13 | 14.8 |
|  | Arthritis | 28.46 | 28.03 | 28.90 | 6.88 | 6.64 | 7.11 | 6.10 | 5.87 | 6.34 | 3.60 | 3.44 | 3.76 | 11.89 | 11.56 | 12.21 | 12.98 | 10.48 |
| **Obesity** |  |  |  |  |  |  |  |  |  |  |  |  |  |  |  |  |  |  |
| Non-obese | No Arthritis | 30.03 | 29.56 | 30.50 | 12.78 | 12.54 | 13.01 | 9.46 | 9.11 | 9.80 | 1.59 | 1.50 | 1.67 | 6.21 | 5.97 | 6.46 | 22.24 | 14.37 |
|  | Arthritis | 27.23 | 26.77 | 27.69 | 6.51 | 6.27 | 6.74 | 6.12 | 5.88 | 6.36 | 3.14 | 2.99 | 3.29 | 11.47 | 11.14 | 11.79 | 12.63 | 9.65 |
| Obese | No Arthritis | 31.29 | 30.51 | 32.07 | 11.5 | 11.17 | 11.84 | 8.78 | 8.27 | 9.28 | 2.29 | 2.15 | 2.43 | 8.71 | 8.22 | 9.21 | 20.28 | 13.79 |
|  | Arthritis | 29.82 | 29.1 | 30.54 | 5.48 | 5.24 | 5.72 | 5.52 | 5.21 | 5.84 | 3.31 | 3.12 | 3.5 | 15.5 | 14.93 | 16.08 | 11.0 | 8.79 |

**Table S4: All health and work life expectancies for females with and without arthritis**

|  |  | |  |  | |  |  |  | |  | |  | |  | | |  | |  |  |  |  |  |  |  |  |  |  |
| --- | --- | --- | --- | --- | --- | --- | --- | --- | --- | --- | --- | --- | --- | --- | --- | --- | --- | --- | --- | --- | --- | --- | --- | --- | --- | --- | --- | --- |
|  | |  | | | Life expectancy, years | | | | | | Healthy working life | | | | | Healthy & not working life expectancy (95% CI) | | | | | Not healthy and & working life expectancy (95% CI) | | | Not healthy & not working life expectancy (95% CI) | | | Healthy life expectancy | Working life expectancy |
|  |  |  |  |  | (95% CI) | | | | | | expectancy, years | | | | |  |  |  |  |  |  |  |  |  |  |  |  |  |
|  |  |  |  |  |  | | | | | | (95% CI) | | | | |  |  |  |  |  |  |  |  |  |  |  |  |  |
| **Overall** | | No Arthritis | | | 33.44 | | 32.94 | | 33.94 | | 11.12 | | 10.91 | | 11.34 | 12.91 | | 12.51 | | 13.31 | 1.67 | 1.58 | 1.75 | 7.74 | 7.44 | 8.04 | 24.03 | 12.79 |
|  |  | Arthritis | | | 32.00 | | 31.69 | | 32.30 | | 6.05 | | 5.90 | | 6.20 | 7.64 | | 7.45 | | 7.82 | 3.12 | 3.01 | 3.22 | 15.19 | 14.93 | 15.45 | 13.69 | 9.17 |
| **Education** | |  | | |  | |  | |  | |  | |  | |  |  | |  | |  |  |  |  |  |  |  |  |  |
| Less than | | No Arthritis | | | 30.07 | | 29.10 | | 31.04 | | 7.22 | | 6.72 | | 7.71 | 12.06 | | 11.29 | | 12.83 | 0.98 | 0.81 | 1.14 | 9.82 | 9.16 | 10.47 | 19.28 | 8.20 |
| high school | | Arthritis | | | 28.90 | | 28.35 | | 29.45 | | 3.72 | | 3.45 | | 3.99 | 6.78 | | 6.58 | | 6.97 | 1.20 | 1.07 | 1.32 | 17.20 | 16.79 | 17.62 | 10.50 | 4.92 |
| High school education | | No Arthritis | | | 34.14 | | 33.54 | | 34.74 | | 11.54 | | 11.30 | | 11.77 | 13.33 | | 12.85 | | 13.81 | 1.75 | 1.65 | 1.84 | 7.53 | 7.17 | 7.88 | 24.87 | 13.29 |
|  |  | Arthritis | | | 32.86 | | 32.49 | | 33.22 | | 6.43 | | 6.25 | | 6.60 | 7.95 | | 7.73 | | 8.17 | 3.44 | 3.32 | 3.56 | 15.04 | 14.74 | 15.34 | 14.38 | 9.87 |
| **Region** | |  | | |  | |  | |  | |  | |  | |  |  | |  | |  |  |  |  |  |  |  |  |  |
| North East | | No Arthritis | | | 33.25 | | 32.07 | | 34.43 | | 11.40 | | 10.88 | | 11.92 | 12.33 | | 11.40 | | 13.26 | 1.47 | 1.28 | 1.65 | 8.05 | 7.30 | 8.80 | 23.73 | 12.87 |
|  |  | Arthritis | | | 32.28 | | 31.54 | | 33.01 | | 6.79 | | 6.60 | | 6.99 | 7.97 | | 7.49 | | 8.46 | 3.62 | 3.37 | 3.86 | 13.89 | 13.25 | 14.54 | 14.76 | 10.41 |
| Midwest | | No Arthritis | | | 34.27 | | 33.24 | | 35.31 | | 11.46 | | 11.02 | | 11.90 | 13.74 | | 12.90 | | 14.58 | 1.54 | 1.37 | 1.70 | 7.54 | 6.93 | 8.15 | 25.20 | 13.00 |
|  |  | Arthritis | | | 31.74 | | 31.13 | | 32.34 | | 6.90 | | 6.58 | | 7.21 | 7.40 | | 7.04 | | 7.76 | 2.93 | 2.73 | 3.13 | 14.51 | 14.00 | 15.01 | 14.30 | 9.83 |
| West | | No Arthritis | | | 33.39 | | 32.21 | | 34.56 | | 10.66 | | 10.12 | | 11.20 | 12.54 | | 11.59 | | 13.49 | 2.26 | 2.01 | 2.50 | 7.94 | 7.19 | 8.68 | 23.20 | 12.92 |
|  |  | Arthritis | | | 33.80 | | 33.09 | | 34.52 | | 6.74 | | 6.16 | | 7.33 | 8.16 | | 7.67 | | 8.66 | 3.71 | 3.32 | 4.10 | 15.19 | 14.47 | 15.90 | 14.90 | 10.45 |
| South | | No Arthritis | | | 32.98 | | 32.18 | | 33.78 | | 10.95 | | 10.61 | | 11.28 | 12.78 | | 12.15 | | 13.41 | 1.67 | 1.54 | 1.80 | 7.58 | 7.12 | 8.04 | 23.73 | 12.62 |
|  |  | Arthritis | | | 31.48 | | 31.02 | | 31.95 | | 6.25 | | 6.01 | | 6.49 | 7.22 | | 6.95 | | 7.49 | 2.81 | 2.66 | 2.95 | 15.21 | 14.81 | 15.60 | 13.47 | 9.06 |
| **Ethnicity** | |  | | |  | |  | |  | |  | |  | |  |  | |  | |  |  |  |  |  |  |  |  |  |
| Black or African American | | No Arthritis | | | 28.49 | | 27.28 | | 29.70 | | 10.04 | | 9.55 | | 10.53 | 9.54 | | 8.69 | | 10.39 | 1.07 | 0.92 | 1.22 | 7.85 | 7.17 | 8.54 | 19.58 | 11.11 |
|  |  | Arthritis | | | 29.36 | | 28.60 | | 30.13 | | 4.73 | | 4.43 | | 5.03 | 5.80 | | 5.44 | | 6.17 | 1.82 | 1.65 | 2.00 | 17.01 | 16.39 | 17.63 | 10.53 | 6.55 |
| White | | No Arthritis | | | 34.02 | | 33.46 | | 34.58 | | 11.29 | | 11.04 | | 11.54 | 13.22 | | 12.77 | | 13.67 | 1.75 | 1.64 | 1.85 | 7.76 | 7.42 | 8.11 | 24.51 | 13.04 |
|  |  | Arthritis | | | 32.47 | | 32.13 | | 32.81 | | 6.29 | | 6.10 | | 6.47 | 7.97 | | 7.76 | | 8.19 | 3.40 | 3.27 | 3.52 | 14.81 | 14.53 | 15.10 | 14.26 | 9.69 |
| **Obesity** | |  | | |  | |  | |  | |  | |  | |  |  | |  | |  |  |  |  |  |  |  |  |  |
| Non-obese | | No Arthritis | | | 33.68 | | 33.17 | | 34.18 | | 11.74 | | 11.47 | | 12.00 | 13.45 | | 13.03 | | 13.87 | 1.49 | 1.39 | 1.58 | 7.00 | 6.70 | 7.30 | 25.19 | 13.23 |
|  |  | Arthritis | | | 31.85 | | 31.50 | | 32.21 | | 6.81 | | 6.61 | | 7.01 | 8.68 | | 8.44 | | 8.91 | 2.70 | 2.59 | 2.82 | 13.67 | 13.38 | 13.95 | 15.49 | 9.51 |
| Obese | | No Arthritis | | | 33.36 | | 32.59 | | 34.14 | | 10.07 | | 9.76 | | 10.38 | 10.42 | | 9.91 | | 10.93 | 2.27 | 2.12 | 2.41 | 10.61 | 10.07 | 11.14 | 20.49 | 12.34 |
|  |  | Arthritis | | | 32.88 | | 32.29 | | 33.47 | | 5.12 | | 4.93 | | 5.31 | 5.87 | | 5.62 | | 6.12 | 3.64 | 3.48 | 3.79 | 18.26 | 17.74 | 18.77 | 10.99 | 8.76 |

**Table S5: All health and work life expectancies for males with and without obesity**

|  |  | Life expectancy, years  (95% CI) | | | Healthy working life  expectancy, years  (95% CI) | | | Healthy & not working life expectancy (95% CI) | | | Not healthy and & working life expectancy (95% CI) | | | Not healthy & not working life ex  expectancy (95% CI) | | | Healthy life expectancy | Working life expectancy |
| --- | --- | --- | --- | --- | --- | --- | --- | --- | --- | --- | --- | --- | --- | --- | --- | --- | --- | --- |
| **Overall** | Not Obese | 29.00 | 28.68 | 29.32 | 10.55 | 10.37 | 10.73 | 7.64 | 7.44 | 7.85 | 2.20 | 2.11 | 2.28 | 8.61 | 8.40 | 8.81 | 18.19 | 12.75 |
|  | Obese | 31.00 | 30.35 | 31.65 | 9.03 | 8.78 | 9.28 | 6.73 | 6.40 | 7.05 | 2.92 | 2.78 | 3.07 | 12.32 | 11.82 | 12.81 | 15.76 | 11.95 |
| **Education** | |  |  |  |  |  |  |  |  |  |  |  |  |  |  |  |  |  |
| Less than  high school | Not Obese | 24.43 | 23.72 | 25.13 | 5.87 | 5.52 | 6.23 | 6.21 | 5.83 | 6.60 | 1.08 | 0.95 | 1.21 | 11.26 | 10.77 | 11.75 | 12.09 | 6.95 |
|  | Obese | 29.47 | 28.32 | 30.61 | 8.71 | 8.16 | 9.26 | 6.59 | 6.01 | 7.18 | 1.25 | 1.03 | 1.47 | 12.91 | 12.03 | 13.79 | 15.30 | 9.96 |
| High school education | Not Obese | 30.07 | 29.68 | 30.45 | 11.30 | 11.09 | 11.50 | 8.07 | 7.82 | 8.31 | 2.39 | 2.29 | 2.49 | 8.32 | 8.08 | 8.56 | 19.36 | 13.68 |
|  | Obese | 31.45 | 30.65 | 32.25 | 9.13 | 8.85 | 9.41 | 6.80 | 6.41 | 7.20 | 3.15 | 2.98 | 3.32 | 12.36 | 11.76 | 12.96 | 15.94 | 12.28 |
| **Region** | |  |  |  |  |  |  |  |  |  |  |  |  |  |  |  |  |  |
| North East | Not Obese | 29.78 | 28.97 | 30.59 | 10.50 | 10.04 | 10.96 | 8.58 | 8.03 | 9.13 | 1.77 | 1.58 | 1.97 | 8.93 | 8.41 | 9.45 | 19.08 | 12.27 |
|  | Obese | 32.36 | 30.82 | 33.91 | 10.79 | 10.16 | 11.42 | 7.82 | 6.96 | 8.68 | 2.60 | 2.27 | 2.93 | 11.16 | 10.04 | 12.27 | 18.61 | 13.39 |
| Midwest | Not Obese | 28.35 | 27.61 | 29.09 | 9.23 | 8.83 | 9.62 | 8.27 | 7.81 | 8.73 | 2.19 | 2.01 | 2.36 | 8.67 | 8.22 | 9.12 | 17.50 | 11.41 |
|  | Obese | 30.15 | 28.92 | 31.38 | 7.28 | 6.80 | 7.76 | 7.05 | 6.42 | 7.68 | 2.47 | 2.18 | 2.75 | 13.35 | 12.38 | 14.33 | 14.33 | 9.75 |
| West | Not Obese | 31.04 | 30.31 | 31.78 | 11.73 | 11.28 | 12.18 | 8.37 | 7.87 | 8.86 | 2.52 | 2.28 | 2.76 | 8.43 | 7.94 | 8.91 | 20.10 | 14.25 |
|  | Obese | 32.53 | 30.83 | 34.23 | 9.01 | 8.28 | 9.74 | 7.19 | 6.34 | 8.05 | 3.92 | 3.47 | 4.37 | 12.41 | 11.07 | 13.74 | 16.20 | 12.93 |
| South | Not Obese | 28.29 | 27.82 | 28.77 | 11.12 | 10.85 | 11.40 | 6.68 | 6.39 | 6.97 | 2.23 | 2.10 | 2.36 | 8.26 | 7.95 | 8.56 | 17.80 | 13.36 |
|  | Obese | 30.14 | 29.11 | 31.17 | 9.34 | 8.96 | 9.72 | 6.03 | 5.53 | 6.52 | 2.71 | 2.50 | 2.92 | 12.06 | 11.29 | 12.83 | 15.37 | 12.05 |
| **Ethnicity** | |  |  |  |  |  |  |  |  |  |  |  |  |  |  |  |  |  |
| Black or African American | Not Obese | 25.21 | 24.34 | 26.07 | 6.68 | 6.29 | 7.07 | 6.25 | 5.77 | 6.73 | 1.35 | 1.18 | 1.52 | 10.92 | 10.33 | 11.51 | 12.93 | 8.03 |
|  | Obese | 29.83 | 28.19 | 31.47 | 9.57 | 8.93 | 10.22 | 7.05 | 6.18 | 7.91 | 1.32 | 1.10 | 1.55 | 11.89 | 10.72 | 13.05 | 16.62 | 10.90 |
| White | Not Obese | 29.65 | 29.29 | 30.00 | 11.37 | 11.16 | 11.58 | 7.82 | 7.59 | 8.05 | 2.37 | 2.27 | 2.48 | 8.08 | 7.86 | 8.30 | 19.19 | 13.75 |
|  | Obese | 31.15 | 30.43 | 31.87 | 9.14 | 8.85 | 9.42 | 6.55 | 6.19 | 6.90 | 3.30 | 3.13 | 3.48 | 12.17 | 11.61 | 12.72 | 15.68 | 12.44 |

**Table S6: All health and work life expectancies for females with and without obesity**

|  |  | Life expectancy, years  (95% CI) | | | Healthy working life  expectancy, years  (95% CI) | | | Healthy & not working life expectancy (95% CI) | | | Not healthy and & working life expectancy  (95% CI) | | | Not healthy & not working life expectancy (95% CI) | | | Healthy life expectancy | Working life expectancy |
| --- | --- | --- | --- | --- | --- | --- | --- | --- | --- | --- | --- | --- | --- | --- | --- | --- | --- | --- |
| **Overall** | Not Obese | 32.89 | 32.60 | 33.17 | 9.64 | 9.48 | 9.80 | 10.23 | 10.03 | 10.44 | 2.00 | 1.93 | 2.08 | 11.01 | 10.80 | 11.22 | 19.88 | 11.65 |
|  | Obese | 33.37 | 32.80 | 33.93 | 7.25 | 7.06 | 7.45 | 6.86 | 6.60 | 7.12 | 3.08 | 2.95 | 3.20 | 16.18 | 15.70 | 16.66 | 14.11 | 10.33 |
| **Education** | |  |  |  |  |  |  |  |  |  |  |  |  |  |  |  |  |  |
| Less than | Not Obese | 29.18 | 28.59 | 29.77 | 5.70 | 5.34 | 6.06 | 9.59 | 9.16 | 10.03 | 0.92 | 0.80 | 1.04 | 12.97 | 12.50 | 13.44 | 15.29 | 6.62 |
| high school | Obese | 30.93 | 29.99 | 31.86 | 4.89 | 4.47 | 5.32 | 6.50 | 6.03 | 6.96 | 1.64 | 1.42 | 1.87 | 17.90 | 17.06 | 18.73 | 11.39 | 6.54 |
| High school education | Not Obese | 33.74 | 33.40 | 34.08 | 10.09 | 9.91 | 10.27 | 10.57 | 10.32 | 10.81 | 2.15 | 2.07 | 2.24 | 10.93 | 10.68 | 11.18 | 20.66 | 12.24 |
|  | Obese | 34.10 | 33.39 | 34.82 | 7.61 | 7.39 | 7.82 | 7.06 | 6.74 | 7.38 | 3.31 | 3.17 | 3.46 | 16.12 | 15.52 | 16.73 | 14.67 | 10.92 |
| **Region** | |  |  |  |  |  |  |  |  |  |  |  |  |  |  |  |  |  |
| North East | Not Obese | 32.93 | 32.24 | 33.62 | 9.84 | 9.45 | 10.23 | 10.24 | 9.73 | 10.75 | 2.21 | 2.01 | 2.41 | 10.65 | 10.14 | 11.15 | 20.07 | 12.05 |
|  | Obese | 33.44 | 32.05 | 34.83 | 6.27 | 5.78 | 6.76 | 7.07 | 6.37 | 7.77 | 3.09 | 2.77 | 3.41 | 17.01 | 15.83 | 18.18 | 13.34 | 9.36 |
| Midwest | Not Obese | 33.00 | 32.43 | 33.57 | 10.08 | 9.75 | 10.41 | 10.42 | 10.00 | 10.84 | 1.89 | 1.74 | 2.04 | 10.61 | 10.19 | 11.03 | 20.50 | 11.97 |
|  | Obese | 32.96 | 31.90 | 34.02 | 7.91 | 7.53 | 8.30 | 7.03 | 6.51 | 7.56 | 2.86 | 2.61 | 3.10 | 15.16 | 14.26 | 16.06 | 14.95 | 10.77 |
| West | Not Obese | 34.25 | 33.59 | 34.91 | 9.06 | 8.67 | 9.46 | 11.21 | 10.69 | 11.72 | 2.21 | 2.01 | 2.40 | 11.77 | 11.25 | 12.30 | 20.27 | 11.27 |
|  | Obese | 33.52 | 32.09 | 34.95 | 6.44 | 5.90 | 6.97 | 6.28 | 5.65 | 6.91 | 4.28 | 3.84 | 4.72 | 16.52 | 15.26 | 17.78 | 12.72 | 10.72 |
| South | Not Obese | 32.12 | 31.68 | 32.56 | 9.45 | 9.20 | 9.70 | 9.74 | 9.43 | 10.05 | 1.88 | 1.77 | 1.99 | 11.05 | 10.72 | 11.38 | 19.19 | 11.33 |
|  | Obese | 33.61 | 32.71 | 34.50 | 7.61 | 7.32 | 7.90 | 6.85 | 6.46 | 7.25 | 2.84 | 2.66 | 3.02 | 16.30 | 15.54 | 17.07 | 14.46 | 10.45 |
| **Ethnicity** | |  |  |  |  |  |  |  |  |  |  |  |  |  |  |  |  |  |
| Black or African American | Not Obese | 28.49 | 27.68 | 29.30 | 8.36 | 7.96 | 8.77 | 7.30 | 6.85 | 7.75 | 1.20 | 1.05 | 1.34 | 11.63 | 11.05 | 12.21 | 15.66 | 9.56 |
|  | Obese | 32.15 | 30.93 | 33.37 | 6.37 | 6.01 | 6.72 | 6.76 | 6.22 | 7.31 | 1.79 | 1.60 | 1.97 | 17.24 | 16.25 | 18.22 | 13.13 | 8.15 |
| White | Not Obese | 33.36 | 33.04 | 33.67 | 9.77 | 9.59 | 9.96 | 10.51 | 10.27 | 10.74 | 2.11 | 2.02 | 2.20 | 10.96 | 10.72 | 11.20 | 20.28 | 11.89 |
|  | Obese | 33.69 | 33.04 | 34.34 | 7.41 | 7.17 | 7.64 | 6.96 | 6.64 | 7.27 | 3.40 | 3.24 | 3.56 | 15.92 | 15.36 | 16.48 | 14.36 | 10.81 |
